# Monkeypox 2022 Outbreak: How Alarming Is the Situation? Epidemiological and Clinical Review

**DOI:** 10.3390/clinpract13010010

**Published:** 2023-01-13

**Authors:** Farah Marraha, Ibtissam Al Faker, Hanane Chahoub, Youness Benyamna, Najlae Rahmani, Salim Gallouj

**Affiliations:** Department of Dermatology, University of Hospital Center of Tangier, Tangier 90000, Morocco

**Keywords:** monkeypox, smallpox, clinical manifestations, diagnosis

## Abstract

Monkeypox is a disease caused by Orthopoxvirus, which also includes the smallpox virus. Several endemics have been reported on the African continent, typically in the western and central regions. However, since 13 May 2022, there have been several cases reported from different member states; the number of confirmed cases in 1 month exceeded the total number of cases reported outside the African continent since the first case in 1970. The World Health Organization (WHO) and Centers for Disease Control (CDC) consider monkeypox as an important disease for global public health. The clinical manifestations and laboratory findings in patients with monkeypox remain unclear. In this brief review, we investigated and compared the different characteristics already reported in cases of monkeypox.

## 1. Introduction

The COVID-19 pandemic has triggered a global health, economic, and political crisis. As a result, the world has become vigilant and attentive to any outbreak. From early May 2022, hundreds of cases of monkeypox, a zoonotic disease endemic to Africa, have been reported in several non-endemic countries. In one month, 1472 cases from 33 member states were documented [1]. The number remains not extremely alarming, despite the spread of the virus outside the African continent, given that, at the beginning of the COVID-19 pandemic, for the same period, the number of cases was much higher (11 March 2020, 7500 cases). However, the World Health Organization (WHO) and Centers for Disease Control (CDC) consider monkeypox as an important disease for global public health. Nevertheless, the risk is moderate, as this is the first time that several cases of monkeypox have been reported in endemic and nonendemic regions [2,3]. As the number of confirmed cases in the early months exceeded the total number of cases reported outside the African continent since the first case in 1970, healthcare workers and concerned organizations must remain vigilant about this infectious disease [4]. Indeed, to date, on 17 December 2022, the spread is considered much more global, with more than 103 non-endemic countries reached and over 81,500 cases reported [5].

In addition to the large number of cases, the particularity of this outbreak lies in the mode of transmission: The cases already reported outside the African continent were related to either the return from a trip to an endemic country or the importation of infected animals [4,6]. However, in the current outbreak, only the first case, reported on 7 May in the UK, was observed from a traveler returning from Nigeria. Since then, several other cases have been confirmed in the UK without any link being established, either with the first case or between subsequent cases [7]. Even in other countries, no evidence of international travel associated with infection has been found, and direct chains of transmission have not been established [4]. This brief review of the literature aimed to report the history and evolution of the epidemiology of human monkeypox and the different clinical manifestations, especially cutaneous manifestations, already reported in other studies, to facilitate and improve detection of this disease. It also aimed to present the modalities of diagnosis and confirmation of the monkeypox virus (MPXV) and the different treatments used and confirmed in the literature. The preventive measures that were already reported in previous outbreaks will ultimately be applied to limit and control the spread of infection.

## 2. History, Epidemiology, and Pathogenesis of Monkeypox

Monkeypox (or mpox, the new preferred term used by WHO) is a disease caused by Orthopoxvirus, which also includes the smallpox virus. The first infected case was reported in 1970 in the Democratic Republic of Congo (DRC) in a 9-month-old baby boy [8]. Subsequently, several endemics have been reported in the African continent, typically in the western and central regions [6]. Breman et al. [9] described for the first time several suspected or confirmed cases reported from 1970 to 1980 in different African countries. A total of 48 cases were identified in six countries: the DRC with the most cases, Cameroon, Côte d’Ivoire, Liberia, Nigeria, and Sierra Leone. Since the 2010s, the number of cases has continued to increase in Africa, more precisely in the DRC [10,11], reaching other countries including Gabon [12] and South Sudan [13]. In 2017, there was also a large outbreak in Nigeria, a country that has not reported a case of monkeypox over the past 39 years [14] (Table 1).

The first case of monkeypox outside Africa was reported in 2003 in the USA, an outbreak of 47 confirmed or probable cases following exposure to animals imported from Ghana [18]. Subsequently, other travel-associated cases were reported in other countries. From September 2018 to November 2021, 11 cases of monkeypox were diagnosed in four non-African countries: one case was reported in Israel in 2018, which originated from Nigeria [19]; another case in Singapore in 2019 [20]; and two cases in the USA (July 2021, Texas; November 2021, Maryland) [21,22]. However, a retrospective study conducted in the UK between August 2018 and September 2021 [17] revealed 11 cases of monkeypox, among which three acquired the virus in the UK: one had the virus nosocomially and two others were infected from household contact (Table 1).

On 7 May 2022, the UK Health Security Agency confirmed a case of monkeypox that had a travel history from Nigeria; other cases were reported to have not left the UK or been in contact with confirmed cases [23]. Since then, several confirmed cases of monkeypox have been reported to the WHO in 33 members states that are not endemic to the MPXV [1]. Nevertheless, the particularity of this outbreak lies in its rapid spread and mode of transmission, and epidemiological investigation must be performed to determine the link between the various reported cases [24].

Monkeypox virus, which presents as a pox-like disease, was isolated for the first time in Denmark in 1958, in monkeys arriving from Singapore that presented a pox-like disease [25] MPXV is a double-stranded DNA virus, measuring 200–250 nm, with a brick-shaped structure. The cell cycle is regulated by proteins encoded by its own genome and occurs in the cytoplasm of infected cells [26].

Based on genetic and geographical data, we distinguished two distinct clades: the West African clade and Congo Basin group clade [27]. The latter seems more virulent, with a high case fatality rate (3.6% and 10.6%, respectively) [28].

After contracting the virus through the respiratory tract (oropharynx, nasopharynx) or close skin-to-skin contact, the virus replicates at the site of inoculation, spreads to local lymph nodes, and then enters the blood circulation to reach other organs. This is the main period of incubation, which lasts 7–21 days [29].

Monkeypox is a zoonotic disease, African rodents are the main reservoir [29], and animal-to-human transmission has been the primary route of spread (Table 2). However, transmission through human sources has also been reported (according to a study, 72.5% and 27.5%, respectively [30]). However, this chain of transmission remains low, with a secondary attack rate varying from 0.3% to 10.2% according to a review of the literature [6].

Moreover, contact with an infected person has not always been established. According to a study [10], among 419 cases, 22% were primary cases.

Human-to-human transmission occurs during close contact with an infected person (body fluids or lesions). This may explain the cases reported in the literature regarding household contacts and persons living in shared housing (e.g., prison) [14,38].

The CDC also reported that airborne contamination is possible and recommended the use of N95 masks and other personal protective equipment when caring for a confirmed case [39].

In this new outbreak, several diagnosed cases were gay, bisexual, or men identifying having had sex with men (MSM) [4,28,33]. Mpox DNA has been detected in seminal fluid, urine, feces, and human saliva [40,41]. However, it is still early to consider a sexually transmitted infection; it may only be related to close contact with genital lesions, skin-to-skin contact, mucosal contact, or introduction of this virus into interconnected social groups [28,33,42].

DNA viruses are more stable and efficient than RNA viruses in detecting and repairing mutations. For this specific outbreak, we cannot discuss mutations to explain the increase in transmission [24]. Public health authorities are working closely with the WHO and CDC to determine the epidemiology, mode of transmission, and possible sources [42].

## 3. Clinical and Laboratory Characteristics and Diagnosis of Monkeypox

The clinical manifestations and laboratory findings in patients with monkeypox remain unclear. Several clinical case studies have been performed to gain deeper insight into the pathology of this viral infection [17,21,22,26,27,43].

In this brief review, we investigated and compared the different characteristics already reported in cases of monkeypox (Table 2).

Based on the (WHO) and (CDC) reports, the national authorities have developed case definition criteria for each outbreak [6,14,33]. However, the clinical presentation of monkeypox resembles that of other viral infections (varicella, herpes, and syphilis, among others); therefore, a definition with greater specificity is required for accurate case detection to avoid unnecessary sampling and to prevent patient confinement and the associated isolation-induced stress, before diagnostic confirmation [44].

### 3.1. Prodrome

The most common prodromal symptoms that precede the skin rash within 1 to 3 days [18], and occasionally up to 2 weeks, include fever, headache, chills, myalgia, and back pain [21]. However, not all patients will present with these symptoms. Yinka et al. [14] observed that fever preceded rash in only 57% of patients. Adler et al. [17] 19 observed this finding in 3/7 patients, and Minhaj et al. [33] in 7/17 patients. Fever is the main criterion for case definition; however, not all patients will have fever, and this fact should be considered during the current outbreak.

### 3.2. Skin Rash: Table 2

A monkeypox rash manifests as a progressive maculopapular to vesicular/pustular rash that gradually undergoes crust formation and finally desquamation over a period of 2–4 weeks, with intact skin between lesions [29,44]. However, this clinical presentation closely resembles that of other illnesses, particularly chickenpox, that are commonly encountered in routine medical practice.

Several studies have reported that the monkeypox rash is clinically characterized by monomorphism (all lesions appear to be at the same stage of development at the time of evaluation) [18,26,44,45]; however, several other reports have refuted these findings [17,18,22,30]. 

The centrifugal distribution and palmoplantar or genital localization of lesions is another feature of this rash [14,26,33,44], which has been described by a recent study during this current epidemic. The rash originated in the genital or perianal area before dissemination in eight patients [33]. However, this presentation was primarily observed in homosexual men and is attributable to close physical contact [46]. Men’s genital lesions may have necrotic crusts or paraphimosis, and the rectal lesions can cause pain on defecation or proctitis [41]. Palmar-plantar involvement is a characteristic finding associated with this infection [42]. Although already been reported, during a suspected monkeypox epidemic, there were several cases of varicella zoster virus (VZV) infection with palmar-plantar involvement [47,48]; however, this localization remains uncharacteristic of VZV infections [44]

### 3.3. Associated Signs and Complications

Lymphadenopathy (axillary, inguinal, and/or cervical) is a prominent sign that differentiates MPXV infection from other similar diagnoses [27,33,44,49]. Lymphadenopathy may occur before or during the rash, with a prevalence of 35–75% [14,18,22,30,33]. Ulcerations may be present in the oral mucosa, tongue, and pharynx, limiting oral intake.

Complications and severe infection, which are known to be associated with the Congo basin clade [3,21] may cause encephalitis, pneumonitis, corneal ulceration, keratitis, and secondary bacterial infections [17,18] Mood disturbance, deep tissue abscesses, and conjunctivitis [17] are the most common complications observed during the present outbreak; however, a few patients may show no complications [33].

The case fatality rate varies based on the viral clades as follows: Central African 10.6% vs. West African 3.6% [6]. To date, 65 deaths have been reported among 82,809 confirmed cases during the current outbreak [50].

Therefore, MPXV infection may present in many forms, and laboratory tests are essential for diagnostic confirmation. However, a study [44] reported that detection of 7/8 of the following 12 specific signs or symptoms serve as useful criteria to initiate specific MPXV investigations: fatigue, being bedridden, nausea, uniform size of lesions, genital distribution, deep-seated firm lesions, distribution on the arms, legs, palms, or soles, lymphadenopathy, conjunctivitis, and fever before onset of rash.

### 3.4. Laboratory Tests and Diagnosis

Isolation of the MPXV DNA using a polymerase chain reaction assay (PCR) is the only method for definitive diagnosis in patients who show a high index of clinical suspicion [25,42,45]. The WHO recommends performing PCR on swabs from lesions surfaces, exudates, or crusts. It also advises collecting and testing additional specimens (such as blood, semen, urine, and genital and rectal swabs) [51]. However, oropharyngeal swabs (OPS) and lesion samples (LS) were evaluated by Ouafi et al., who concluded that OPS might not be helpful for diagnosis [52].

During the current outbreak, we performed genomic analysis of MPXV isolates from Portugal and Belgium and compared these with all available MPXV sequences; we observed that this particular organism was more closely associated with the virus identified in the West African clade [53]. Immunological methods (enzyme-linked immunosorbent assay) may be used for immunoglobulin (Ig)G and IgM antibody detection. In principle, these antibodies are detected in the serum after the onset of the skin eruption, approximately 5 days for IgM, and up to 8 days for IgG [26]. MPXV can also be visualized using electron microscopy, although this method is not useful for diagnostic confirmation and only shows that the virus belongs to the Poxviridae family [54]. Concomitant sexually transmitted infections (STI) were reported in several studies, in 29% of cases according to Thornhill et al. [35], and in 17% according to Tarin et al. [36], most commonly chlamydia and syphilis in addition to human immunodeficiency virus (HIV). Laboratory tests to look for these STIs are recommended [35].

## 4. Management Treatment

Most reports in the literature describe that patients usually recover spontaneously after a few weeks without specific treatment [9,14,18,30,33]. However, some antiviral agents have been used in severe cases during previous outbreaks [17,18,19,20,21].

Tecovirimat (also known as TPOXX) is an antiviral that blocks the intracellular release of the virus and was approved by the Food and Drug Administration after the CDC confirmed its safety; it has been licensed by the European Medicines Agency [26,55]. In animals, studies have shown that tecovirimat treatment, when compared to placebo treatment, improves survival from deadly monkeypox virus infections at various illness phases [56]. Tecovirimat is also shown to reduce lesions and length of hospitalization; however, this agent is not approved for post-exposure prophylaxis [17,57]. According to the CDC’s Emergency Access Investigational New Drug Protocol, tecovirimat may be used to treat infections caused by non-variola orthopoxviruses, such as monkeypox [58] (Table 3). It is also available through a randomized controlled clinical trial called STOMP (Study of Tecovirimat for Human Monkeypox Virus) [59].

Brincidofovir inhibits viral DNA synthesis and has shown antiviral activity against MPXV in vivo [60]. This drug was administered to three patients in the UK in 2018 and led to a transient reduction in the viral PCR cycle; however, its use was associated with an increase in liver enzyme levels [17]. Brincidofovir has shown a modest survival benefit even in MPXV-infected dogs (29% vs. 14%) [61]. Cidofovir has the same efficacy but may have more side effects than Brincidofovir [62].

Most patients affected during the current outbreak were between 20 and 50 years of age, which is attributable to the loss of cross-protective immunity with the smallpox vaccine, since it was discontinued worldwide in the 1980s [24,42]. Reportedly, the smallpox vaccine offers 80–95% protection against MPXV [63]. The WHO and CDC [64,65] recommend the use of JYNNEOS™ (Bavarian Nordic, Copenhagen, Denmark) and ACAM2000^®^ (Emergent Product Development Gaithersburg, Inc., Rockville, MD, USA) for the prevention of mpox disease, as they are considered safer and more effective options. JYNNEOSTM and ACAM2000^®^ differ in a number of ways. While JYNNEOSTM is a replication-deficient modified vaccinia Ankara virus, ACAM2000^®^ is a replication-competent vaccinia virus. Therefore, ACAM2000^®^ causes a greater skin reaction at the inoculation site than JYNNEOSTM. In addition, according to recommendations, immunosuppressed individuals must avoid the use ACAM2000^®^ [66]. Currently, the WHO does not recommend vaccination against monkeypox for the general public; however, it does advise vaccination for at-risk populations, including healthcare workers, laboratory technicians, and men who have sex with men (MSM). For contacts of cases, post-exposure preventative vaccination (PEPV) is suggested, ideally within four days of the initial exposure [64].

The other preventive measures include patient isolation in a single room in a hospital or at home and instructing the patient to wear a mask. The use of personal protective equipment (PPE) by healthcare workers is recommended [67]. Quarantine for forward-traced contacts has been proposed to control the spread of infection [67], but the WHO recommends it only if the symptoms are present [68]. Each country, under the guidance of the WHO or CDC, must develop strategies to avoid mpox spread, and these plans should concentrate on case discovery, management, contact tracing, laboratory investigation, isolation, and immunization [67,68]. Most infections in the present outbreak are caused by MSM. Therefore, health messaging and delivery strategies may need to be adapted to specifically address these individuals, for example, through specific websites, dating applications, or media programs. 

This brief summary may aid comprehension of the clinical and epidemiological aspects of monkeypox; however, our review has some limitations. First, it is difficult to describe all the clinical cases reported in the literature, especially when attempting to compare past and present outbreaks. Second, since this is a recent outbreak, we can regularly expect additional articles that clarify and update the epidemiological and clinical information.

**Table 3:** The different treatments used for the management of monkeypox.

Treatments	Formulations and Dosing	Criteria for Use	Contraindications	Adverse Events	Monitoring and Drug Interactions
Tecovirimat [58]	PO, IV * 200 mg capsule(s) weight-based dosing taken by mouth with a full glass of water within 30 min after eating for adult: 600 mg twice daily for 14 days	− Severe disease (hemorragic, large number of lesions, sepsis, encephalitis) − Involving functionally sensitive anatomic area: pharynx, vulva, vagina, or rectum − Patient immunocompromised, pediatric populations, pregnant or breastfeeding − Skin conditions inferred to increase risk for infection	− Severe renal impairment (CrCl < 30 mL/min) − Allergy to ingredients	Oral: nausea, abdominal pain, vomiting, headache IV: infusion site reactions	Repaglinide: Monitor blood glucose for hypoglycemic symptoms Midazolam: Monitor for effectiveness of midazolam
Brincidofovir [69]	PO: Adult > 48 kg: 200 mg once weekly for two doses	− Have severe disease OR are at high risk for progression to severe disease − Lack of improvement while receiving tecovirimat or recrudescence − Contraindication for oral or IV tecovirimat	None	− Nausea, abdominal pain, vomiting − Increased serum transaminases and serum bilirubin	Consider alternative medications that are not OATP1B1 or 1B3 inhibitors to avoid increased brincidofovir -associated adverse reactions
Cidofovir [69]	IV: 5 mg/kg once weekly for 2 weeks, followed by 5 mg/kg IV once every other week	Same indication as brincidofovir; brincidofovir may have an improved safety profile over cidofovir	-Allergy to ingredients -Serum creatinine > 1.5 mg/dL; CrCl ≤ 55 mL/minute; use with or within 7 days of nephrotoxic agents	− Hypotony of eye, iritis, uveitis, nephrotoxicity, fever, − Decreased serum bicarbonate	Probenecid, Nephrotoxic agents: discounted at least 7 days before prior to starting therapy with cidofovir

* Approved in May 2022 [58]; PO: per os; IV: intravenous.

## 5. Conclusions

Monkeypox virus causes a clinical presentation closely resembling that of other illnesses. Several clinical case studies are needed to gain deeper insight into the pathology of this viral infection. Climate change, deforestation, movement of people globally, important travel spread, and the loss of cross-protective immunity with the smallpox vaccine are probably the main factors responsible for the importance of this pandemic. Several epidemiological investigations must be performed to determine the link between the various reported cases.

## Figures and Tables

**Table 1:** Number of Monkeypox cases reported in the previous outbreak.

Country/Region	1970–1979 *	1980–1989 *	1990–1999 *	2000–2009 *	2010–2020 *	2021 **
DRC	38	343	511	10,027	18,788	4594 **
Nigeria	3	—	—	—	181	98
Liberia	4	—	—	—	6	--
Cameroon	1	1	—	—	3	--
Côte d’Ivoire	1	1	—	—	—	--
Sierra Leone	1	—	—	—	2	--
Gabon	—	4	9	—	—	--
Central African Republic	—	8	—	—	61	--
Congo	—	—	—	73	24	--
South Sudan	—	—	—	19	—	--
United States	—	—	—	47	—	2 ***
United Kingdom	—	—	—	—	4	3 ****
Israel	—	—	—	—	1	-
Singapore	—	—	—	—	1	-

DRC: Democratic Republic of Congo; * [6]; ** [15]; *** [16]; **** [17].

**Table 2:** Clinical and demographic characteristics of cases reported in previous outbreaks and 2022 outbreak from the available articles.

Author, Year (Citation)	Study Year/ Period	Country/Region	Confirmed Cases	Age Case Report/Age Median	Transmission	Clinical Presentation	Localization	Complication	Mortality Rate	Treatment/ Vaccination
Breman, 1980 [9]	1970–1979	DRC Nigeria Liberia Côte d’Ivoire Sierra Leone	38 3 4 1 1	7 months–41 y 35 y 4–9 y 5 y 24 y	Animal to human and Human to human	Simultaneously: papules, vesicles, and pustules→ umbilication→ drying, and desquamating. (process 2–4 weeks) about 0.5–1 cm Lymphadenopathy 18 cases: submandibular, cervical and inguinal	-Peripheral distribution, including the palms and soles 23 (49%) cover the entire body Mucous membranes: tongue and genitalia	1 case/47: Corneal lesion caused unilateral blindness	8/47 (17%) died: between 7 months and 7 y	(9%): vaccination scar
Jezek, 1988 [30]	1981–1986	DRC	338	4.4 (3 months–69 y)	Animal to human: suspected in 245 cases (72.5%); Human to human: in 93 cases (27.5%)	Monomorphic (all lesions at the same stage): 79% Pleomorphic: 21% Discrete: 58.5% Semiconfluent: 31.5% Confluent: 10% Haemorrhagic: 0% Lymphadenopathy: 50% (duration 2–4 weeks)	**Body distribution:** Centrifugal: 83.5% Centripetal: 4.5% Indefinite: 12% Facial: 87% Palmar: 70% Plantar: 66.5% **Mucous membranes:** Oral: 70% Conjunctival: 19% Genital: 30%	In 39.5%: of cases Secondary bacterial infection of skin: **34 cases** Bronchopneumoni pulmonary distress: **34 cases** Vomiting/diarrhoadehydration: **22 cases** Keratitis, corneal ulceration **11 cases** Septicaemia: **1** Encephalitis: **1**	33/338 (10%) between: 3 months and 8 y	(13%): vaccination scar * this study analyzed only the clinical characteristics of unvaccinated cases 222
Aplogan, 1997 [31]	1997	DRC	419	85% < 16 y	Animal to human Human to human	Vesicular-pustular rash with five or more facial pock marks Cervical lymphadenopathy (69%), sore throat (63%), mouth ulcers (50%), cough (41%), and diarrhea (11%)	NR	NR	case fatality ratio: 1.5% from 4 to 8 y	NR
Huhn et al., 2005 [18]	2003	United States	34	70.6% (>18 y) 29.4% (<18 y) median age: 26 y	Animal to human Human to human	Cutaneous rash (papules, vesicles, pustules 65%; umbilicated 33.8%; macules 30%) Ulcerated or necrotic lesions: 25% Hemorrhagic pustules: 5% Monomorphic: 67.7% pleomorphic: 29% No rash: 3.2% Fever: 85% (median duration 8 days) Chills: 71% Headache: 65% Myalgias: 56% Adenopathy: 71%	Localized: 25.8% Centrifugal: 48.4 Centripetal: 3.23 Even distribution: 22.6% Arms/hands: 81.3% Legs/Feet: 65.6% Head/Neck: 62.5% Chest/Abdomen: 56.2% Back: 46.9% Palms: 28.1% Groin/Buttocks and Soles: 9.4% Mucosa: 6.3%	Adults: bacterial su-perinfection keratitis and corneal ulceration, Children: encephalopathy and retropharyngeal abscess.	0	21% vaccinated
McCollum, 2015 [32]	2011–2014	DRC	3	28 y M 24 y M 23 y F	Likely all animal to human	Fever followed by a rash: umbilicated pustules surrounded by an area of erythema and some crusts (yellow and black)	Starting on the face and mouth and then extending to the arms, trunk, and legs	NR	0	NR
Yinka et al. [14]	2017–2018	Nigeria	122	2–50 y 84 (69%) were male	Human to human 36%: epidemiologically linked with a confirmed case	Vesiculo-pustular Rash: 100% Oral ulcer: 32% Fever: 79% Headache: 73% Pruritus: 69% Myalgia: 63% Throat: 58% Lymphadenopathy: 63%	Face: 96% Legs: 91% Trunk: 56% Arms 79% Palms: 69% Genitalia: 68% Soles and feet: 64%	Conjunctivitis: 23% Vomiting nausea: 21% Spontaneous abortion	7 deaths (case fatality rate 6%)	NR
Adler et al. [17]	2018–2021	UK	7	4: 30–40 y 1: 40–50 y 1 < 2	NR	Vesicular pustular, papular macular rash Sub-ungual lesion From 10 to 150 lesions 4/6 lymphadenopathy	Face trunk: 7/7 Limbs: 3/7 Scalp: 2/7 Palms and/or soles: 4/7 Genital: 5/7	Ulcered inguinal lesion:2/7 Depp tissue abscesses:1/7 Conjunctivitis 1/7	None	3 patients treated: oral **Brincidofovir** (7 days post- rash) 200 mg qw 1 patient: **tecovirimat** 600 mg b.I.d for 2 weeks
Costello [22]	2021	Maryland, USA	1	28-year-old	Travel-associated Monkeypox case from Nigeria (human to human suspected)	Vesicle rash Umbilicated pustules on erythematous base 2–4 mm erosions in oral mucosa and pustules mucosal lips Cervical lymphadenopathy	Acrofacial pustules, first the propagation in arms, trunk, and inner thighs Oral mucosa and lower mucosal lip	0	0	Acyclovir IV
Minhaj et al. [33]	2022	USA	17		14/17 International travel associated; 11 different countries during 21 days preceding	Cutaneous rash: vesicles and pustules Lymphadenopathy 8/17	8/17 started in the genital or perianal area All but one patient developed a disseminated rash, occurring on the arms, trunk, legs, and face	NR	NR	NR
Antinori et al. [34]	2022	Italy	4	median age: 30	All reported international travel and MSM	Asynchronous rash lymphadenopathy; fever, asthenia	Genital-perianal area, then suprapubic area Chest, feet, legs, and back	1/4 Inguinal lymphadenitis	0	1 case reported vaccination 30 y earlier
Thornhill et al. [35]	2022	16 countries	528	median age: 38 male: 527	Travel history: 28% MSM: 95%	Skin lesions: 95% Of which 58% vesiculopustular Fever Lymphadenopathy Anorectal pain Pharyngitis	Ano-genital area (73%); trunk, arms, or legs (55%); face (25%); and the palms and soles (10%)	1 case: epiglottitis 2 cases myocarditis	0	5% received specific treatment cidofovir tecovirimat and vaccinia immune globulin
Tarín-Vicente et al. [36]	2022	Spain	181	median age: 37 male: 97%	Travel history: 14% MSM: 92%	Pustular lesions: 90% Vesicular lesions: 26% Influenza-like illness: 81%	Anogenital > 90% Oral ulcer: 25% Hands and feet: 60%	proctitis and tonsillitis	0	6 patients: Topical cidofovir
Selb et al. [37]	2022	Germany	521	median age: 38 male: 100%	Travel history: 26/521 MSM: 259/521	NR	NR	NR	NR	NR

DRC: Democratic Republic of Congo; y: years; NR: not reported; MSM: men identifying having sex with men; IV: intravenous.

## Data Availability

Not applicable.
